# Motivational Interviewing for encouraging quit attempts among unmotivated smokers: study protocol of a randomized, controlled, efficacy trial

**DOI:** 10.1186/1471-2458-12-456

**Published:** 2012-06-19

**Authors:** Delwyn Catley, Kari Jo Harris, Kathy Goggin, Kimber Richter, Karen Williams, Christi Patten, Ken Resnicow, Edward Ellerbeck, Andrea Bradley-Ewing, Domonique Malomo, Robin Liston

**Affiliations:** 1 Department of Psychology, University of Missouri-Kansas City, 5100 Rockhill Road, Kansas City, MO 64110, USA; 2 School of Public and Community Health Sciences, The University of Montana, 32 Campus Drive, Skaggs Rm 352, Missoula, MT 59812, USA; 3 Department of Preventive Medicine & Public Health, University of Kansas Medical Center, 3901 Rainbow Boulevard, MS 1008, 4004 Robinson, Kansas City, KS 66160, USA; 4 Department of Biomedical and Health Informatics, University of Missouri – Kanas City School of Medicine, 2411 Holmes Street, Kansas City, MO 64108, USA; 5 Department of Psychiatry and Psychology, Mayo Clinic, 200 First Street SW, Rochester, MN 5590, USA; 6 Department of Health Behavior and Health Education, School of Public Health, University of Michigan, 1415 Washington Heights, Ann Arbor, MI 48109-2029, USA

**Keywords:** Smoking, Motivational Interviewing, Health education, Brief advice

## Abstract

**Background:**

Although the current Clinical Practice Guideline recommend Motivational Interviewing for use with smokers not ready to quit, the strength of evidence for its use is rated as not optimal. The purpose of the present study is to address key methodological limitations of previous studies by ensuring fidelity in the delivery of the Motivational Interviewing intervention, using an attention-matched control condition, and focusing on unmotivated smokers whom meta-analyses have indicated may benefit most from Motivational Interviewing. It is hypothesized that MI will be more effective at inducing quit attempts and smoking cessation at 6-month follow-up than brief advice to quit and an intensity-matched health education condition.

**Methods/Design:**

A sample of adult community resident smokers (N = 255) who report low motivation and readiness to quit are being randomized using a 2:2:1 treatment allocation to Motivational Interviewing, Health Education, or Brief Advice. Over 6 months, participants in Motivational Interviewing and Health Education receive 4 individual counseling sessions and participants in Brief Advice receive one brief in-person individual session at baseline. Rigorous monitoring and independent verification of fidelity will assure the counseling approaches are distinct and delivered as planned. Participants complete surveys at baseline, week 12 and 6-month follow-up to assess demographics, smoking characteristics, and smoking outcomes. Participants who decide to quit are provided with a self-help guide to quitting, help with a quit plan, and free pharmacotherapy. The primary outcome is self-report of one or more quit attempts lasting at least 24 hours between randomization and 6-month follow-up. The secondary outcome is biochemically confirmed 7-day point prevalence cessation at 6-month follow-up. Hypothesized mediators of the presumed treatment effect on quit attempts are greater perceived autonomy support and autonomous motivation. Use of pharmacotherapy is a hypothesized mediator of Motivational Interviewing’s effect on cessation.

**Discussion:**

This trial will provide the most rigorous evaluation to date of Motivational Interviewing’s efficacy for encouraging unmotivated smokers to make a quit attempt. It will also provide effect-size estimates of MI’s impact on smoking cessation to inform future clinical trials and inform the Clinical Practice Guideline.

**Trial registration:**

ClinicalTrials.gov NCT01188018

## Background

Tobacco use is the primary cause of preventable diseases in the United States. In 2009, an estimated 46.6 million adults in the United States were current smokers [1]. Smoking in the US accounts for about 443,000 deaths yearly, approximately 5.1 million in years of potential life lost, and $96.8 billion in productivity losses [2]. Reducing the prevalence of smoking remains one of the country’s most important public health goals [1,2].

To reduce the health and social consequences of tobacco use, it is essential to reduce the decades-long lag between initiation of regular smoking and cessation. Many smokers will smoke for 20–30 years before quitting [3]. The sooner a smoker stops, the greater the gains in life expectancy [4]. Although most smokers are “interested” in quitting smoking [5], approximately 40% are not planning on quitting in the next 6-months and another 40% have no plans to quit in the next month [6]. Unfortunately, established clinical smoking cessation interventions focus on those in the remaining 20% that are ready and seeking assistance to quit. For example, many tobacco quit-lines in the United States, require callers to be ready to quit before comprehensive services are provided [7]. Likewise, primary care physicians are less likely to counsel patients to quit, or refer them to counseling, if they believe the patient is not ready to quit [8,9]. Given those not ready to quit comprise a large majority of the smoking population, proactive intervention with less motivated smokers could have a significant public health impact, even if such intervention had only moderate efficacy [10].

One promising approach to encourage cessation among less motivated smokers is Motivational Interviewing (MI), a treatment approach that emerged from the alcohol and drug treatment literature that focuses on fostering motivation for, and commitment to behavior change. MI has been defined as a collaborative, person-centered form of guiding to elicit and strengthen motivation for change [11]. Principles of MI include using a collaborative style, eliciting individuals’ reasons for change rather than persuading, and supporting autonomy so that individuals do not feel pressured to change and can feel autonomously or “internally” motivated. These principles are manifested in communication methods (e.g., open-ended questions, affirming, and reflective listening) that are used to strategically elicit and enhance the individual’s elaboration of “change talk” (statements in the direction of making a change) and increase their awareness of the discrepancy between their current behavior and their perception of what would be ideal behavior. These strategies are thought to be more effective than questioning, persuading, or giving advice.

Meta-analyses have indicated that MI-based interventions have modest positive effects on smoking cessation relative to interventions such as brief advice to quit [12-14]. However, the Clinical Practice Guideline and several meta-analyses have noted significant deficiencies in the evidence base [12,13,15]. Significant limitations of the existing literature include the inadequate evidence of intervention fidelity, the lack of research comparing MI to alternative interventions of equal intensity, and the lack of focus on the role of motivation to quit and motivation and quit attempts as outcomes [12,13]. Evidence of fidelity to MI principles is essential for internal validity and because null effects could be due to “Type 3 error” (i.e., low quality implementation rather than ineffective treatment), yet prior MI studies have rarely included the use of a validated instrument for assessing MI adherence [12].

Equally important for advancing the research on MI is whether the positive effects observed in prior studies can be attributed to the benefits of MI or simply to the greater duration of contact with participants. Teasing out counseling approach effects from general attention effects is critical given the cost and complexity of implementing an MI intervention. For example, studies have shown that significant training and practice is necessary for MI to be properly implemented [16] and there is a lack of research comparing MI with potentially more straightforward but equally intensive interventions (e.g. intensive health education) [12].

A third major concern with the existing evidence base for MI is the lack of attention to the role of motivation. Motivation mediates cessation outcomes and, according to meta-analysis, MI may be more effective for low motivated smokers [17]. Accordingly, there is also evidence that cognitive-behavioral skills training may be more effective than MI for smokers already motivated to make a quit attempt [18]. Taken together, these findings suggest that tailoring counseling style to motivational level may be most effective.

The purpose of the present study is to conduct a randomized controlled trial to examine the efficacy of MI for inducing quit attempts among low motivated smokers while addressing key limitations of prior studies. The primary outcome will be the effect of MI on any quit attempt by the 6-month follow-up relative to an intensity-matched control condition (Health Education; HE) and minimal intervention control condition (Brief Advice to quit; BA). The secondary outcome is biochemically confirmed 7-day point-prevalence cessation rates at 6-month follow-up. Participants’ perceived autonomy support from their counselor and autonomous motivation for quitting will be examined as mediators of any treatment effect on quit attempts. Participants who choose to set a quit date for a quit attempt will be offered pharmacotherapy and pharmacotherapy use will also be examined as a mediator of MI’s effect on cessation. In addition, the goal is to implement the MI intervention with high fidelity verified with independent coders using a validated coding scheme. The study uses quit attempts rather than cessation as the primary outcome to reduce the needed sample size. The study aims to be a preliminary step toward a much larger trial that will also include more highly motivated participants for comparison and focus on smoking cessation as the main outcome.

## Methods/Design

### Trial design

This is a multi-arm parallel group, randomized trial with imbalanced randomization (2:2:1 for MI, HE, and BA, respectively) being conducted in the United States. Blinding of counselors is not feasible in this study. Participants are informed that they will be randomly assigned to one of three counseling approaches that differ in style and/or number of sessions. Although participants can differentiate whether they are in the BA versus MI or HE because of the different number of sessions, they are not informed in any way regarding the names, the nature, or distinctions between HE and MI and therefore will be blind to which of these two treatments they receive.

### Recruitment, participants, and setting

The study is being conducted at a single university-based site in Kansas City, Missouri. The study protocol is in compliance with the Helsinki Declaration and was reviewed and approved by the Institutional Review Board of the University of Missouri – Kansas City (#0978). Smokers are recruited community-wide through word of mouth, newspaper ads, flyers, billboards, internet advertising, and physician referral using printed cards. Recruitment materials invite participation in a study “for smokers” or “smokers not quite ready to quit” and provide a study phone number to call for more information. Potential participants who enquire about the study are informed that the goal is to learn how best to talk to smokers about their health. They are informed that although their smoking will be discussed they will not be required to quit. Eligibility criteria are being 18 years or older, English speaking, reporting smoking a minimum of 1 cigarette per day, having a mailing address and telephone number, willingness to participate in all study components, not currently pregnant or planning to become pregnant in the next 6 months, not currently using a smoking cessation medication, not planning to move from the metropolitan area in the next 6 months, and not currently motivated or ready to quit smoking (defined as scoring 6 or less on a 0–10 point scale of motivation to quit smoking and having no intention to quit in the next 7 days). Potential participants are pre-screened by phone and those likely to be eligible are scheduled for a baseline visit at which time they are re-screened and enrolled if eligible. After the first 24 participants were enrolled, confirmation of self-reported smoking status using a carbon monoxide monitor (Bedfont Scientific piCO + Smokerlyzer®) was added to the re-screen. Eligibility is determined by a CO level of 7 ppm or higher [19,20].

### Trial interventions

#### Motivational interviewing

MI consists of 4 sessions (baseline, week 6, week 12 and week 18) of approximately 20 minutes each conducted in-person (baseline and week 12) and over the phone (week 6 and week 18). The 4-session schedule is altered should participants set a quit date. All remaining sessions are scheduled on the day after the selected quit day and then every week after that. Although the optimal number of sessions of MI is not known [12,17] four sessions were chosen as a practical compromise between having too many sessions for participants who do not progress toward making a quit attempt and having enough sessions to both motivate and then assist smokers who do progress to making a quit attempt.

In MI counselors assist participants to explore and resolve ambivalence regarding quitting smoking, consistent with MI principles and strategies described by Miller & Rollnick [21]. MI emphasizes a collaborative, evocative, and autonomy supportive counseling approach, using specific communication skills (open-ended questions, affirmations, reflections, and summaries) to express empathy, develop discrepancy between client goals/values and current behavior, increase change talk, and “roll with” statements of resistance to change. MI sessions are characterized by a collaborative, interactive counseling style in which participants are engaged in the process of thinking and talking about their smoking behavior through the counselors’ use of strategic reflective listening and occasional open-ended questions and summaries. In MI, providing information on health-risks and the benefits of quitting is minimized and offered by counselors only when the participant asks for or appears clearly interested in the information.

Participants who express interest in quitting receive a self-help guide for quitting and, consistent with MI methods for strengthening commitment for change, are encouraged to develop a 5-step plan for quitting (set a quit date, change environmental triggers, prepare for triggers and urges, reward yourself, and use medication) based on the Clinical Practice Guideline [15]. However, to remain consistent with MI this step is delivered in an MI style (i.e., maintaining the principles and specific communication skills that characterize the MI approach). For example, counselors continue to support patient autonomy, use reflective listening and encourage the participant to identify potential solutions before offering their own suggestions or giving advice. Participants who choose to set a quit date within the period of their planned participation in the study are offered a 12-week supply of free pharmacotherapy for smoking cessation (described in more detail below).

Meta-analyses have indicated that manualized MI interventions may produce poorer outcomes [14,17] so MI is implemented without a structured guideline for the flow of the session (with the exception of being required to have the participant consider all 5 of the elements of the plan to quit should the participant express interest in quitting). Counselors are trained to use the various tools and strategies of MI as they see fit.

#### Health education

HE is designed to provide a plausible alternative intervention to MI that is equivalent in contact time and that can be delivered with fidelity. To match MI there are 4 HE sessions of approximately 20 minutes conducted on the identical schedule and in the same format (phone versus in-person) as MI. For participants who set a quit date the schedule is altered in the same fashion as in the MI arm.

In HE counselors deliver health education designed to persuade participants to quit. The method of motivating participants is based on providing a strong rationale for quitting based on the relevant risks of smoking, rewards of quitting, and addressing the roadblocks to quitting. These elements are consistent with the Clinical Practice Guideline, which recommends the use of the “5 R’s” (i.e., discuss patient relevant risks of smoking, rewards of quitting, roadblocks to quitting, and repeat at each visit), but excludes the Guideline’s recommendation to use MI principles in their delivery. To ensure HE is distinct from MI, counselors in this arm follow a semi-structured script and use printed slides (when counseling is in-person) to deliver the intervention.

The 5-part counseling protocol begins with an assessment of participants’ smoking and quitting history and their experience of common symptoms from smoking. Counselors point out the link between participants’ current symptoms and their smoking. In the absence of symptoms counselors underscore that the best thing that participants can do for their health is to quit smoking. Second, counselors describe major long-term (e.g., heart-disease) and short-term (e.g., slower wound healing) risks of smoking (4–5 at each session) and provide education on one of a variety of smoking related topics (i.e., content of cigarettes, costs of smoking, the addictive nature of tobacco, luring of smokers by tobacco companies, and the dangers of second-hand smoke). Third, counselors discuss the potential rewards of quitting including short and long-term health benefits. Fourth, counselors ask about participants’ roadblocks to quitting and provide suggestions or counterarguments for each. Finally, counselors provide personalized advice underscoring the importance of cessation and ask participants if they are interested in making a plan to quit. HE is designed to be warm and supportive (i.e., not confrontational) but differs from MI in that the primary focus is on giving information rather than eliciting participant engagement in considering their smoking behavior. Participants are engaged with a few specific questions that are used to guide counselors in providing appropriate information (e.g., suggestions for overcoming the specific roadblocks mentioned by the participant).

As with the MI arm, participants who express interest in quitting receive a self-help guide for quitting, and are encouraged to develop a 5-step plan for quitting based on the Clinical Practice Guideline [15]. Participants who choose to set a quit date within the period of their planned participation in the study are offered a 12-week supply of free pharmacotherapy for smoking cessation.

#### Brief advice

BA is designed to mimic usual care and provide a comparison treatment that is consistent with that used in many prior MI studies for smokers [12,22]. The design of BA is based on the recommendations of the Clinical Practice Guideline [15]. Participants meet with a counselor for approximately 5 minutes during which they are asked about common smoking related symptoms and are provided with clear, strong, personalized advice to quit. Smokers are then asked if they are interested in quitting and, if so, are provided with a self-help guide to quitting and asked about their planned quit date. Participants who choose to set a quit date within the period of their planned participation in the study are offered a 12-week supply of free pharmacotherapy for smoking cessation.

#### Provision of pharmacotherapy

All participants who set a quit date are offered pharmacotherapy to assist with their quit attempt to meet standard of care. Due to evidence of its potentially superior efficacy [23-25], counselors encourage the use of varenicline (Chantix®) when not contra-indicated. Participants who cannot or who do not wish to use varenicline are offered nicotine replacement therapy in the form of the patch or the lozenge. To avoid free medication serving as the primary motivator for smokers to quit, medication is not mentioned or offered to participants until after they have demonstrated a commitment to make a quit attempt by setting a quit date.

Participants who decide to use varenicline are provided with a 12-week supply (a starter pack and 2 continuation packs). Participants who choose chose the patch receive an 8 week supply if they smoke less than 10 cigarettes per day (14 mg for 6 weeks, 7 mg for last 2 weeks), a 10 week supply if they smoke 10 to 39 cigarettes per day (21 mg for 6 weeks, 14 mg for 2 weeks, 7 mg for 2 weeks), and a 10 week supply if they smoke 40 or more cigarettes per day (42 mg for 6 weeks, 28 mg for 2 weeks, and 14 mg for 2 weeks). Participants who choose the lozenge receive a 12-week supply (2 mg if they smoke their first cigarette 30 minutes after waking and 4 mg if they smoke within 30 minutes of waking). Participants are given instructions for the proper use of their medications at the time that medications are dispensed. Participants who decide to use medications during a phone counseling session are scheduled for a brief medication-dispensing visit. Participants with cautions for using varenicline (e.g., history of depression) are monitored closely for adverse events by means of weekly phone calls.

#### Interventionists and training

Three Master’s level health professionals with prior training and experience using MI are trained to deliver all 3 interventions to eliminate any potential of confounding across arms due to counselor specific effects. The use of dedicated, specifically trained staff (as opposed to nurse or physician providers for example) is necessary because of the complexity of delivering all three of these treatments with high fidelity. While this diminishes generalizability of the results to primary care, meta-analyses suggest that the effects of MI are at least similar [13], if not improved, when delivered by general practitioners [12].

Training for MI consisted of refresher training in the practice and principles of MI through reading materials, video demonstrations, and a half-day training workshop; training in the MI protocol for the study (i.e., procedures and requirements for implementing MI in this study); and completion of role-play practice sessions with feedback from the MI arm supervisor in a group setting (i.e., with all counselors present). Counselors then practiced the MI protocol with pilot participants and received group supervision until they met criterion on rating scales designed for fidelity monitoring (described below) for 3 consecutive sessions.

Training for HE and BA was similar, including training in the HE and BA protocols for the study (i.e., procedures and content for implementing HE and BA in this study) and completion of role-play and pilot participant practice sessions accompanied by group supervision until 3 consecutive sessions met criterion.

#### Fidelity assurance procedures

All counseling sessions in the study are digitally recorded. Counselors alternate in receiving regular group supervision by separate expert supervisors (weekly for MI, every other week for HE, and monthly for BA). For each supervision session an audio-file is either randomly selected from those completed by the counselor since their last supervision session, or the counselor identifies a challenging session that they wish to receive feedback on. The supervisor provides verbal feedback as the group listens to the session and rates adherence to the respective protocols using rating scales adapted for this study. The rating scales assess procedural and content requirements for each arm. For example, the MI rating scales assess completion of basic procedures (e.g., remind the participant that the session is being audio recorded) and fidelity on 13 elements of MI (e.g., uses reflective listening, rolls with resistance vs. confronting, affirms and builds efficacy) and 1 global rating (how well did the counselor conduct the session). HE and BA rating scales similarly assess fidelity on basic procedures and key counseling elements (e.g., providing personalized advice, recommending quitting, linking participants’ symptoms to smoking). To externally verify that MI and HE sessions differ as expected, 10% of the MI and HE sessions (excluding those sessions that involve making quit plans which are expected to be quite similar) are randomly selected for coding on adherence to MI principles using the Motivational Interviewing Treatment Integrity code [26] by an expert independent coding group. Coders are blind to the study arm of the session and the study hypotheses. We anticipate that MI sessions will receive scores of 4 or higher on the 1–5 global rating and be significantly higher than HE sessions on ratings of evocation, collaboration, empathy, and autonomy support. With respect to frequency measures of counselor behavior we expect MI sessions to have a higher reflection to question ratio and significantly fewer instances of giving information.

### Randomization

Prior to starting enrollment a computer-generated randomization sequence was created by the project statistician that determines whether subjects will receive MI, HE, or BA in accordance with the 2:2:1 allocation ratio. The statistician used sequentially numbered opaque envelopes to conceal the group assignment sequence until randomization. Once eligibility is confirmed participants complete the informed consent process. The research assistant then opens the next numbered envelope in the sequence to reveal the participant’s group assignment and the participant begins the computerized baseline survey. In this manner all study personnel remain blind to the condition to which the participant is assigned until after participant’s eligibility is determined and informed consent is obtained.

### Measures

Participants complete computer-administered assessments at baseline, week 12, and 6-month follow-up. Baseline variables include socio-demographic characteristics (gender, age, education, employment), smoking characteristics (cigarettes smoked per day, number of years smoking, number of prior quit attempts), level of nicotine dependence using the Severity of Dependence Scale [27], and single item measures of motivation and confidence to quit [28-30]. The primary outcome measure is the occurrence of any quit attempt defined as a serious quit attempt of at least 24 hours [31] by 6-month follow-up. The secondary outcome is biochemically verified 7-day point-prevalence abstinence at 6-month follow-up [32,33]. Biochemical verification is conducted by research staff using cotinine test strips for saliva [34]. Hypothesized mediators of MI’s effect on quit attempts are autonomous motivation and autonomy support which are assessed using the Treatment Self-Regulation Questionnaire [35] and the Health Care Climate questionnaire [36,37], respectively. For those who decide to make a quit attempt, weeks of pharmacotherapy use is assessed with a self-report checklist [29] and will be examined as a mediator of MI’s effect on cessation.

### Reimbursement

Participants receive compensation for time and travel in the form of payment for each survey and counseling session completed (i.e., $30 for completing baseline and week 12 surveys, $50 for the 6-month follow-up surveys and $10 for each counseling session). Total compensation is up to $120 for BA and $150 for MI and HE.

### Analyses

#### Power and sample size

Sample size calculations are based on planned comparisons of quit attempts between MI and HE, and MI and BA, and based on conservative (i.e., high) estimates of a 15% and 30% quit attempt rate in the BA and HE arm respectively. The calculations are based on a two-tailed test with an alpha of .05 and a power level of 80%, and an estimated attrition rate of 25%. A sample of 102 per group is necessary to detect a difference of 25% in the proportion of subjects making a 24 hour quit attempt between MI and HE groups, and a sample of 51 per group is necessary to detect a difference of 40% between MI and BA groups. To conserve resources, the study is not powered for all three pairwise comparisons allowing for a smaller BA sample size and the 2:2:1 allocation ratio. For the same reason, the study focuses on building motivation and generating quit attempts and is not powered for the secondary aim of determining group differences in 7-day point prevalence abstinence. The trial aims to obtain effect-size estimates for the MI intervention relative to HE and BA on cessation that will inform future studies. Based on the 2:2:1 allocation ratio the target sample is therefore 255 (102 MI, 102 HE, and 51 BA).

#### Data analyses

All analyses will be conducted in an intent-to-treat manner. Our primary analyses will utilize a planned comparisons approach to compare the proportion of those making at least one quit attempt for HE and MI, and for BA and MI, using logistic regression. If preliminary analyses comparing groups on baseline variables suggest potential confounding effects by any covariate, these will be included in the logistic regression models. For the secondary hypotheses we will also use logistic regression to compare the effectiveness of HE and MI, and MI and BA, on 7-day point prevalence abstinence at follow-up. To evaluate autonomy support and autonomous regulation as mediators of the presumed effect of MI on quit attempts, and weeks of pharmacotherapy use as a mediator of the presumed effect of MI on cessation, we will follow the procedural steps outlined by Baron and Kenny [38] using logistic regression and the Sobel test [39,40].

## Discussion

Although MI is a promising tool for healthcare providers who regularly encounter low motivated smokers, stronger evidence for its efficacy is needed to justify the additional training and maintenance of skills needed for this approach. This study is designed to provide the most rigorous evaluation of MI to date, focusing specifically on smokers not motivated to quit and examining quit attempts as the primary outcome. Examination of quit attempts rather than cessation conserves resources but will provide a strong preliminary indication of the potential efficacy of MI for tobacco treatment in this population. A much larger sample size would be necessary to power a trial in which cessation is the primary outcome. However, the secondary outcome of smoking cessation at 6-month follow-up in this study will provide an effect size estimate that can inform the design of future MI trials focused on cessation. On the other hand, should MI fail to prove more efficacious than brief advice or health education, it would encourage reconsideration of the current Clinical Practice Guideline and prompt further research to develop more powerful intervention methods to motivate smokers to try to quit.

## Abbreviations

MI: Motivational interviewing; HE: Health education; BA: Brief advice.

## Competing interests

Varenicline (Chantix ®) was provided by Pfizer through Investigator Initiated Research Support (No. WS759405). Pfizer reviewed a draft of the manuscript but played no role in the design of the study, data collection, the writing of the manuscript, or the decision to submit for publication.

Drs. Catley, Goggin, and Resnicow regularly conduct Motivational Interviewing training.

## Authors’ contributions

DC led the overall conception, design, and implementation of the study, led the design and implementation of the MI intervention, and drafted the manuscript. KJH assisted in the overall conception, design and implementation of the study, led the implementation of the data collection and project management system, and helped to draft the manuscript. KG assisted in the overall conception, design, and implementation of the study and co-led the design and implementation of the Health Education and Brief Advice interventions, and helped revise the manuscript. KR assisted in the overall conception, design, and implementation of the study and co-led the design of the Health Education and Brief Advice interventions, and helped revise the manuscript. KW assisted in the overall conception and design of the study and led the design and implementation of the statistical aspects of the study. CP assisted in the overall conception, design and implementation of the study and helped revise the manuscript. KR assisted in the overall conception and design of the study and helped revise the manuscript. EE assisted in the design and implementation of the medical aspects of the project and helped revise the manuscript. ABE assisted in the conception, design and implementation of the counseling aspects of the study. NM assisted in the conception, design and implementation of the counseling aspects of the study. RL assisted in the design and implementation of the counseling aspects of the study. All authors read and approved the final manuscript.

## Pre-publication history

The pre-publication history for this paper can be accessed here:

http://www.biomedcentral.com/1471-2458/12/456/prepub
